# General Rehabilitation Program after Knee or Hip Replacement Significantly Influences Erythrocytes Oxidative Stress Markers and Serum ST2 Levels

**DOI:** 10.1155/2022/1358858

**Published:** 2022-03-30

**Authors:** Maciej Idzik, Jakub Poloczek, Bronisława Skrzep-Poloczek, Elżbieta Chełmecka, Jerzy Jochem, Dominika Stygar

**Affiliations:** ^1^Independent Public Health Care, Opole Cancer Centre prof. Tadeusz Koszarowski, Katowicka 45-061 Street, 46-020 Opole, Poland; ^2^Department of Rehabilitation, 3rd Specialist Hospital in Rybnik, Energetyków 46 Street, 44-200 Rybnik, Poland; ^3^Department of Internal Medicine, Diabetology, And Nephrology, Faculty of Medical Sciences in Zabrze, Medical University of Silesia, Katowice, Poland; ^4^Department of Physiology, Faculty of Medical Sciences in Zabrze, Medical University of Silesia, Jordana Street 19, 41-808 Katowice, Poland; ^5^Department of Statistics, Department of Instrumental Analysis, Faculty of Pharmaceutical Sciences in Sosnowiec, Medical University of Silesia, Ostrogórska 31 Street, 41-200 Katowice, Poland

## Abstract

The survival of erythrocytes in the circulating blood depends on their membranes' structural and functional integrity. One of the mechanisms that may underlie the process of joint degeneration is the imbalance of prooxidants and antioxidants, promoting cellular oxidative stress. The study is aimed at observing the effects of the 21-day general rehabilitation program on the erythrocytes redox status and serum ST2 marker in patients after knee or hip replacement in the course of osteoarthritis. Erythrocytes and serum samples were collected from 36 patients. We analyzed the selected markers of the antioxidant system in the erythrocytes: catalase (CAT), glutathione reductase (glutathione disulfide reductase (GR, GSR)), total superoxide dismutase activity (SOD), glutathione peroxidase (GPx), glutathione transferase (GST) activity, and cholesterol and lipofuscin (LPS) concentration. In serum, we analyzed the concentration of the suppression of tumorigenicity 2 (ST2) marker. After the 21-day general rehabilitation program, the total SOD and GPx activity, measured in the hemolysates, significantly increased (*p* < 0.001) while LPS, cholesterol, and ST2 levels in serum significantly decreased (*p* < 0.001). General rehabilitation reduces oxidative stress in patients after knee or hip replacement in the course of osteoarthritis. Individually designed, regular physical activity is the essential element of the postoperative protocol, which improves the redox balance helping patients recover after the s4urgery effectively.

## 1. Introduction

Osteoarthritis (OA) is the most common form of arthritis, one of the most common joint disorders in the world [1]. Osteoarthritis of the knee and hip is a common condition in the elderly [1]. Since no effective treatment options for osteoarthritis or osteoarthritis modifying therapies exist, joint replacement remains the only treatment [2]. However, OA pathogenesis and the accompanying pain are two commonly researched therapeutic targets that, if approached correctly, should help prevent disease progression and relieve the patients from pain [2].

Regardless of its type, postoperative rehabilitation significantly reduces pain, increases the range of motion and muscle strength, and limits the use of pain medications [3, 4]. However, strenuous exercise increases oxygen uptake and energy requirements, which intensifies the mitochondrial energy metabolism and promotes the formation of free radicals and oxidative stress [5]. Cellular oxidative stress resulting from the imbalance of prooxidants and antioxidants is thought to be one of the mechanisms that may underlie the process of joint degeneration [6].

Erythrocytes are one of the many cell types in which a redox imbalance can occur. Fatty acids in the membranes, elevated oxygen levels, and the presence of hemoglobin inside make erythrocytes a natural target for free radicals [7]. The oxidative processes in the erythrocytes entail membrane injury, modifications of the membrane's structural and functional elements, and changes in the membrane architecture. All these processes cause an increase in erythrocytes' mean osmotic fragility and inhibition of Mg^2+^ and Ca^2+^ ATPase activity [7]. The survival of erythrocytes in the circulating blood plasma depends on the structural and functional integrity of their membranes, which determines the mechanical behavior of cells. Erythrocytes express several physiological defenses against intracellular oxidative stress, including cellular antioxidant enzymes: superoxide dismutase (SOD), catalase (CAT), glutathione reductase (GR), glutathione peroxidase (GPx), and glutathione S-transferase (GST) [7, 8]. Reactive oxygen species (ROS), such as superoxide radicals, hydrogen peroxide, and especially hydroxyl radical, are toxic for cells. They modify amino acid residues and oxidize sulfhydryl groups in proteins, break peptide bonds, remove metals in metalloproteins, depolymerize nucleic acids, cause point mutations, and also oxidize polysaccharides and polyunsaturated fatty acids. Protection against toxic and mutagenic ROS is provided by enzymes of antioxidative defense, with SOD and CAT being the most important of them [9].

The intensity of oxidative stress can be assessed using various general and specific indicators [10]. Lipofuscin (LPS) as a product of unsaturated fatty acid oxidation is considered one of them. Lipofuscin formation is directly related to an increased concentration of other oxidative stress markers. Reactive oxygen species are too short-lived to be detected directly. However, they react with lipids and produce lipid peroxidation products that may serve as indirect biomarkers of *in vivo* oxidative stress status and related diseases. Malondialdehyde (MDA) is one of the principal and most studied low-molecular-weight end products of lipid peroxidation. The high cytotoxicity of MDA results from its ability to bind proteins or nucleic acids [11]. Malondialdehyde (MDA) cross-links erythrocytes' phospholipids and proteins which impairs a variety of membrane-related functions and leads to their diminished survival and death [12].

Dysregulation of ST2/IL-33 signaling and suppression of tumorigenicity 2 (ST2) marker production is observed in various inflammatory diseases such as cardiac disease [13–16], inflammatory bowel disease (IBD) [17–20], graft-versus-host disease (GVHD) [21–28], and type-2 diabetes [29–32]. ST2 was first discovered to function as a mediator of type 2 inflammatory responses [33]. Targeting ST2 demonstrated protective effects in the respiratory [34], skin [35], and kidney [36] diseases and autoimmune neurological pathologies [37].

Pain and disability are the two most frequent OA complications, which lead to a significant reduction in a patient's physical activity. The decreased physical capacity and sedentary lifestyle negatively influence the heart muscle condition, leading to comorbid heart diseases [38]. The American Heart Association and American College of Cardiology indicated ST2 as an important factor of heart failure: it predicts hospitalization and death in patients and, together with natriuretic peptides, shows a prognostic value [39]. Our previous study reported that a 21-day general rehabilitation significantly improved patients' physical efficiency and exercise capacity after hip or knee replacement [40]. Rheumatic diseases are chronic inflammatory disorders in which the immune system attacks itself and the body's organs [41]. A growing number of studies have demonstrated a critical role of the IL-33/ST2 axis in rheumatic diseases, including systemic lupus erythematosus (SLE), rheumatoid arthritis (RA), primary Sjögren's syndrome (pSS), systemic sclerosis (SSc), psoriatic arthritis (PsA), gout, and ankylosing spondylitis (AS), indicating a promising potential for IL-33/ST2-targeting therapy in rheumatic disease [41]. Although the alterations in the blood induced by physical training influence changes in other tissues, the effects of physical training-induced oxidative stress on blood have not been extensively investigated. However, changes in erythrocytes' properties can lead to impaired oxygen transport and result in cell and tissue hypoxia [42]. Our previous work presented significant changes in the plasma parameters related to the 21-day postoperative general rehabilitation [40]. Since, selected in this work, oxidative stress markers and also ST2 are important and sensitive indicators of inflammatory, prooxidative, and cardiac status, we aimed to observe the effects of 21-day general rehabilitation program on the erythrocytes' redox status and changes in ST2 serum levels in patients after knee or hip surgical replacement in the course of osteoarthritis.

## 2. Materials and Methods

### 2.1. Ethical Statement and Permissions

The study was approved by the Ethics Committee of the Medical University of Silesia in Katowice (KNW/002/KB1/106/17 issued on 03/10/2017) and followed the Declaration of Helsinki guidelines. Every participant of the study was informed about the study protocol, its benefits, and possible risks. All participants returned the written informed consent before the study started.

### 2.2. Study Group

Patients were recruited in the outpatients' clinic and the Department of Rehabilitation, 3rd Specialist Hospital in Rybnik, 2017-2018. The study included patients that in the prior 6 months underwent hip or knee arthroplasty due to advanced degenerative disease. The study excluded any patients with other inflammatory or immune disease, cancer, ischemic heart disease, heart, kidney, or liver failure.

Upon the arrival to the outpatients' clinic, each patient was initially examined and interviewed. The initial examination consisted of the resting electrocardiogram (ECG) and blood pressure measurement. The body mass and height measurements were also recorded. The interview is aimed at identifying patients with inflammatory disorders, infections, renal or hepatic insufficiencies, active coronary artery disease, diabetes, heart failure, hormonal replacement therapy, or supplementation with antioxidants. All patients with the above-mentioned health deficiencies occurring 3 months before the study were excluded from the study.

Eventually, the study included 41 patients after total hip (*n* = 29; 71%) or knee (*n* = 12; 29%) replacement. Twenty-two of them were men, and 19 were women (54% and 46%, respectively), aged 61.0 ± 8.1 years on average. The day the study started, they were 89.6 ± 9.7 days after the replacement surgery. Five patients were excluded from the oxidative stress markers analyses due to health conditions diagnosed during the study course.

### 2.3. General Rehabilitation Program

A 21-day general rehabilitation program started ca. 90 days after knee or hip endoprosthesis implantation. The program constituted physiotherapy, daily living activities training, and education on nutrition. The daily sessions were conducted for 45 min, from 8:00 to 8:45 a.m. The individual rehabilitation program consisted of aerobic walking (30-45 min), strength training (20-30 min), rotor/bicycle training (30-45 min), and a cool-down phase (15 min). The patients were asked to continue the activities at home to sustain the effects at the beneficial level [43]. The responsible physiotherapist individually adjusted the type of exercises (strength and balance exercises) and training modalities (number and sets of repetitions, duration of resting time) for each patient and then monitored their progress.

Before starting and after completing the 21-day general rehabilitation program, each patient underwent the 6-minute walk test (6MWT) [44] to track changes in the patient's functional exercise capacity and assess the effectiveness of the rehabilitation program [45, 46]. The results of the 6-minute walk test (6MWT) after a 21-day general rehabilitation in the studied patients were presented in Skrzep-Poloczek et al. [40].

### 2.4. Blood Collection

Blood samples (5 mL) were collected from the ulnar vein the day before the first and the day after the last rehabilitation session (in the morning, at 8:00 a.m, before breakfast). The blood was collected to the standard blood tubes with EDTA (1.6 mg/mL EDTA-K3) and into tubes with a clot activator (S-Monovette, SARSTEDT, AG & Co. KG, Numbrecht, Germany).

The samples for serum analysis were centrifuged at 1500 g for 10 min at 4°C, transferred into 1 mL cryotubes, and stored at −80°C for later analyses. The pellet was washed with PBS buffer (0.01 M, 0.14 M NaCl, and pH 7.4) three times, and the separated erythrocytes were cooled to 4°C and then also stored at −80°C for later analysis. Before the analysis, erythrocytes were thawed, diluted with distilled water, and then again cooled to 4°C.

### 2.5. Oxidative stress markers, cholesterol, and ST2 analysis

To assess the status of the antioxidant system in the erythrocytes, we determined the activity of catalase (CAT), glutathione reductase (glutathione disulfide reductase (GR, GSR)), total superoxide dismutase activity (SOD), glutathione peroxidase (GPx), and glutathione transferase (GST). Additionally, we analyzed lipofuscin (LPS), cholesterol concentration in the erythrocytes, and suppression of tumorigenicity 2 (ST2) marker concentration in blood serum, to assess the intensity of the patients' oxidative stress during the rehabilitation program.

#### 2.5.1. Catalase (CAT) Activity (EC 1.11.1.6)

CAT activity was determined using the Aebi method [47]. In short, the hemolysate homogenate was mixed with the TRIS/HCl buffer (50 mM, pH 7.4). The reaction was initiated with freshly prepared H_2_O_2_. The hydrogen peroxide decomposition rate was measured spectrophotometrically at 240 nm. CAT activity was expressed as units per 1 g of hemoglobin (IU/g Hb).

#### 2.5.2. Glutathione Reductase (GR) Activity (EC 1.8.1.7)

GR activity was determined using the kinetic method [48]. The changes in absorbance, resulting from the changes in NADPH concentration after reaction with oxidized glutathione, were measured at 340 nm. GR activity was expressed as *μ*mol of NADPH utilized in 1 min per 1 g of hemoglobin (IU/g Hb).

#### 2.5.3. Superoxide Dismutase (SOD) Activity (EC 1.15.1.1)

Total SOD activity was determined using the Oyanagui method [49]. In short, xanthine oxidase catalyzes the production of superoxide anion that with hydroxylamine produces nitroso ion. The nitroso ion reacts with n-(1-naphthyl)ethylenediamine and sulfanilic acid, and the concentration of the product of this reaction can be measured spectrophotometrically at 550 nm. The total SOD activity was presented as nitrite units (NU) per mg of hemoglobin, where one NU represents 50% blockage of nitrite ions formation [49].

#### 2.5.4. Glutathione Peroxidase (GPx) Activity (EC 1.11.1.9)

Glutathione peroxidase (GPx) was measured using Paglia and Valentine's kinetic method [50], recording the decrease of absorbance at 340 nm. The GPx activity was expressed in U/g Hb, where one unit of enzyme activity (U) represents the amount of enzyme causing oxidation of 1 *μ*mol NADPH in 1 min at 25°C.

#### 2.5.5. Glutathione S-Transferase (GST) Activity (EC 2.5.1.18)

GST activity was determined using the Habig and Jakoby kinetic method [51]. In short, the reaction mixture with reduced glutathione was added to hemolysate samples, and after initial stabilization, 1-chloro-2,3-dinitrobenzene was added. The changes in absorbance were measured at 340 nm for at least 3 min. The GST activity was expressed as *μ*mol of thioether formed within 1 min per 1 g of hemoglobin (IU/g Hb).

#### 2.5.6. Lipofuscin (LPS) Concentration

LPS concentration was determined as described by Tsuchida et al. [52]. The serum was mixed with ethanol-ether (3 : 1; *v*/*v*), shaken, and centrifuged. The fluorescence intensity was measured at 345 nm (for absorbance) and 430 nm (for emission) in a dissolved solid. LPS concentration was expressed in relative lipid extract fluorescence (RF), where 100 RF corresponds to the fluorescence of 0.1 *μ*g/mL of quinidine sulfate in 0.1 N sulfuric acid. The inter- and intra-assay coefficients of variations (CV) were 2.8% and 9.7%, respectively.

#### 2.5.7. Total Cholesterol Concentration

Total cholesterol was measured in erythrocytes membrane lipid extracts using a commercial enzymatic assay (Waco Chemicals GmbH, Neuss, Germany) based on Allain et al.'s methodology [53]. The assay detection limit was 1.8 mg/dL. The intra- and interassay precision were <1.1% for both indicators. The results are expressed as milligram of total membrane cholesterol per deciliter.

#### 2.5.8. Suppression of Tumorigenicity 2 (ST2) Marker Concentration

Suppression of tumorigenicity 2 (ST2) marker concentration was determined using the Quantikine Human ST2/IL-1 R4 Immunoassay kit (R&D Systems, Minneapolis, MN, United States). The assay was calibrated according to the manufacturer's recommendations. The values were normalized to a standard curve. ST2 concentration was determined using the Cobas analyzer (Cobas 6000e501, Roche Diagnostics, Mannheim, Germany). The test sensitivity was 13.5 pg/mL, and the assay range was 31.3-2000 pg/mL.

#### 2.5.9. Protein Concentration

Hemoglobin concentration in hemolysates was determined using the modified Drabkin method [54].

### 2.6. Statistical Analysis

Statistical analysis was performed using STATISTICA 12.5 PL (StatSoft, Cracow, Poland). The mean value ± SD (for a normal distribution) and median with lower–upper quartile range (for data with skewed or nonnormal distribution) were chosen to express the interval data. The Shapiro–Wilk test and the quantile-quantile plot evaluated the distribution of variables. The homogeneity of variances was checked with Levene's test. The Mann–Whitney *U*-test, the nonparametric Kruskal–Wallis test, or the two-way parametric ANOVA with *post hoc* contrast analysis was used for data comparison. The data with skewed distribution were log-transformed before analysis. A *p* < 0.05 was considered statistically significant, and all the tests were two-tailed.

## 3. Results

The study included 41 patients after hip or knee replacement surgery. The mean age of the patients was 59.0 ± 7.0 years (range: 40-72 years). Women constituted 46.3% of the study group. There were no statistically significant differences in the age of the patients depending on their sex (*p* = 0.859). The location of lesions depending on the age of the patients was also analyzed, and no significant differences were found in both groups (*p* = 0.978) (Table 1).

### 3.1. General Health Indicators

Our previous work presented the biochemical and morphological characteristics of the blood of patients before and after the 21-day general rehabilitation program [40]. Here, we assessed whether the biochemical parameters analyzed in the patients' serum changed compared to the laboratory norm [55].

### 3.2. C-Reactive Protein (CRP)

Before starting the 21-day rehabilitation program, all patients showed no signs of infection, and their health status was confirmed by CRP parameter < 5 mg/L. However, after completing the 21-day rehabilitation program, this parameter showed even lower values, as it decreased significantly in 80% of patients (Table 2).

The applicable standards of erythrocyte sedimentation rate (ESR) for women and men take into account their age. Reference values for women < 60 years are 3-15 mm/h, for women ≥ 60 years are 3-20 mm/h, for men < 60 years are 1-10 mm/h, and for men ≥ 60 years are 1-15 mm/h. Analysis of the results showed that before the 21-day rehabilitation program, the ESR values were above the norm in six patients (1 woman and 5 men). After the 21-day rehabilitation program, the ERS values fit within the norm for female patients, while in 3 men, the ESR values were above the norm (Table 2).

### 3.3. Glucose

Glucose concentration in the range of 3.9-5.5 mmol/L is considered normal. Before the therapy, 13 patients had elevated glucose levels, and after the therapy, elevated glucose levels were noted for only 6 patients. Values below the norm, both before and after the 21-day rehabilitation program, were observed only in 1 patient.

### 3.4. Creatinine

The reference values for creatinine in healthy individuals are 60-120 *μ*mol/L for men and 50-110 *μ*mol/L for women. The creatinine levels fit within the norm in all patients, both before and after the 21-day rehabilitation program.

### 3.5. Platelets

The platelet count of 150-400 K/*μ*L is considered normal. The platelet count was below the norm before the therapy for one patient, and it slightly increased after the 21-day rehabilitation program. The platelet count was within the normal range of healthy individuals in the remaining patients both before and after the 21-day rehabilitation program.

### 3.6. Hematocrit

All women had hematocrit within the normal range (35–45%) before and after the 21-day rehabilitation program. However, the hematocrit of 7 male patients before the rehabilitation was below the lower limit of its norm (the normal range for male patients is 40*–*50%). After the rehabilitation, only 5 male patients presented hematocrit values below the norm.

### 3.7. Red Blood Cells (RBC)

The norm of red blood cells count is 3,500,000-5,200,000/*μ*L (3.5–5.2 M/*μ*L) for women and 4,200,000-5,400,000/*μ*L (4.2–5.4 M/*μ*L for men. Before the 21-day rehabilitation program, 5 men had low RBC count, while after the rehabilitation, the RBC count was low in three male patients. As for female patients, 3 women had a high RBC count before the rehabilitation, while after the 21-days rehabilitation program, the RBC count was normal in all female patients.

### 3.8. Hemoglobin (HGB)

The normal hemoglobin range for women is 12-16 g/dL and 14-18 g/dL for men. Low HGB levels were noted in 11 male patients before and 9 male patients after the 21-day rehabilitation program. The HGB levels in female patients fit within the normal limits before and after the 21-day rehabilitation program.

### 3.9. White Blood Cells (WBC)

The reference norm value of white blood cells count is 4,000-10,000 leukocytes/mm^3^. The elevated leukocyte count was observed only in one patient before the rehabilitation started, and all study participants presented normal WBC count after the rehabilitation concluded.

### 3.10. Total Cholesterol

The norm for cholesterol level for healthy individuals is <190 mg/dL. Only 4 patients presented normal cholesterol levels before the rehabilitation started, and only 5 after it concluded. Although most patients presented elevated cholesterol levels, it is worth noting that in 78% of patients (32 individuals), the cholesterol level decreased after the 21-day rehabilitation program.

### 3.11. High-Density Lipoprotein (HDL)

The norm for high-density lipoprotein is 40-80 mg/dL for women, and 35-70 mg/dL for men. Thirteen female patients had low HDL levels before the rehabilitation started, and nine women after it. Only one male patient presented a low HDL level before the rehabilitation. In the whole study group, the decreased HDL level was observed in 11 patients (male and female together).

### 3.12. Triglycerides (TG)

Triglyceride reference values are as follows: correct result < 150 mg/dL. Before the 21-day rehabilitation program, 18 patients (44%) had normal TG levels, and after the rehabilitation concluded, the normal TG levels were observed in 26 patients (63%).

### 3.13. Low-Density Lipoprotein (LDL)

The normal level of low-density lipoprotein (LDL) is <115 mg/dL. The correct LDL concentration was noted only in 3 patients (7%) before the rehabilitation started and 5 patients (12%) after the rehabilitation concluded. A decrease in LDL concentration in the course of the 21-day rehabilitation program was found in 11 patients (27%).

### 3.14. Oxidative Stress Markers, Cholesterol, and ST2

A 21-day rehabilitation program significantly influenced the levels of selected oxidative stress markers: SOD and GPx assessed in erythrocytes hemolysates, as well as LPS, cholesterol, and ST2 assessed in serum obtained from patients after knee or hip replacement. CAT, GR, and GST activities were the same as before the 21-day rehabilitation program (Table 3).

After a 21-day rehabilitation program, the total SOD activity increased on average by 63.5 ± 23.7 NU/mg Hb (95% CI: 55.4-71.6) and GPx activity increased by 11.2 ± 8.2 NU/g Hb (95% CI: 8.5-14.0). On the contrary, LPS concentration in the serum decreased by 86.6 RF (95% CI: 24.9-84.4, cholesterol levels decreased by 16.4 ± 19.3 mg/dL (95% CI: 9.9-23.0), and the ST2 level decreased by 0.2 ± 0.1 ng/mL (95% CI: 0.1-0.2).

## 4. Discussion

Our previous work presented biochemical and morphological characteristics of the blood of patients before and after the 21-day general rehabilitation program [40]. We reported that the individually designed general rehabilitation positively affected the patients' blood glucose and lipids concentrations. Glucose, total cholesterol, LDL, and TG were significantly lower, and HDL levels were significantly higher when compared to their initial levels. Also, we observed that C-reactive protein, platelets, and hematocrit were lower after the rehabilitation proving that general rehabilitation helped reduce inflammation and prevent clot formation [40].

Hypoxia leads to decreased antioxidant defense in muscles and other organs. The decreased activity of superoxide dismutase (SOD) and lower glutathione levels have been observed under hypoxic conditions [42]. Oxygen generates reactive oxygen species (ROS) production necessary for cell signaling processes. Nevertheless, ROS also exerts detrimental effects on organs function including the heart's function [42]. In this study, we assessed the impact of postoperative rehabilitation on oxidative stress markers, cholesterol, and ST2 measured in erythrocytes of patients after hip or knee replacement surgery in the course of osteoarthritis. We analyzed catalase (CAT), glutathione reductase (glutathione disulfide reductase (GR, GSR)), total superoxide dismutase (SOD), glutathione peroxidase (GPx), and glutathione transferase (GST) activities, lipofuscin (LPS), and cholesterol concentration in the erythrocytes, as well as ST2 concentration in serum. To the best of our knowledge, this is the first study designed to compare the oxidative stress markers, cholesterol, and ST2 marker, measured in erythrocytes or serum, before and after the general rehabilitation cycle. Our results show a significant increase in SOD and GPx activity in erythrocytes and a significant decrease in ST2 marker concentration in the serum. Simultaneously, we observed a reduction in LPS and total cholesterol concentration in patients' erythrocytes. A previous study on patients with hip or knee replacement enrolled into the general rehabilitation program [40] showed that physical rehabilitation improves serum oxidative stress parameters and positively affects patients' general health. The patients showed improved glucose and lipid profiles and inflammation and blood clotting parameters [40]. They also presented greater physical efficiency and exercise capacity, as determined by the 6-minute walk test (6MWT). After the rehabilitation process, the patients walked a longer distance in 6MWT, but their body mass remained the same [40]. Osteoarthritis affects patients' antioxidative and oxidative metabolism by significantly impairing antioxidant defense [56]. Antioxidants ensure the proper functioning of the organism by neutralizing reactive oxygen species. In mature erythrocytes, SOD, CAT, and GPx participate in this process [57]. They control ROS production and limit or repair damage caused by them [58]. The interventional antioxidants, also known as free radical scavengers, creating the antioxidant barrier [59] include SOD, CAT, and GPx. They remove ROS from the cellular and intercellular spaces and extracellular fluids, such as plasma, lymphatic fluid, cerebrospinal fluid, and articular synovial fluid [60]. The family of SOD enzymes comprises three isoforms with different structural characteristics of the prosthetic group (containing Mn or Cu and Zn), compartmentalization, and functional significance. In mature erythrocytes, the Cu/Zn isoform (SOD1) catalyzes the superoxide anion dismutation. The superoxide anion is formed mostly by Hb autoxidation. The produced hydrogen peroxide is degraded to oxygen and water by CAT and GPx. In nonpathological conditions, GPx degrades most of the H_2_O_2_ by oxidizing GSH into GSSG [57]. Here, we confirm that total SOD and GPx activity in erythrocytes increased after regular, individually adjusted physical activity during 21-day general rehabilitation after hip or knee replacement. Our previous study showed that total SOD activity measured in patients' serum also increased after 21 days of general rehabilitation. The effects of 21-day general rehabilitation, adjusted to the patient's abilities, designed and controlled by a physiotherapist, were compatible with results obtained by other research groups and resulted from the oxidative stress after physical training [40]. Oxidative stress is an essential factor that modulates the progression of complications triggered by physical activity implemented as a therapy. Teixeira et al. [61] demonstrated that the erythrocyte SOD activity was higher in kayakers and canoeists. Evelson et al. [62] determined that resting SOD activity in well-trained rugby players was higher than men with a sedentary lifestyle. Melikoglu et al. [63] also reported that SOD and GPx activities were higher in basketball players than in men with a sedentary lifestyle. Other researchers showed that GPx activity is associated with the maximal oxygen uptake attained by specific training, and its increased activity reflects adaptation to the exercise-induced oxidative stress [64]. Here, we observed the same tendency in total SOD and GPx activity in erythrocytes of patients who underwent knee or hip replacement and were enrolled in a 21-day controlled and individualized therapeutic physical activity. Moderate physical activity, appropriately matched with the fitness level of post hip or knee surgery patients, leads to significantly increased exercise capacity and induces multiple physiological changes in the body including changes in blood biochemistry and metabolism [40]. Physical activity increases blood oxygenation, tissue perfusion, and oxygen metabolism leading to mild oxidative stress [42]. Mild oxidative stress induced by physical activity may evoke adaptive changes, such as increased expression of antioxidant enzymes causing a general improvement in the function of tissue antioxidative systems [42].

Reactive oxygen species oxidize proteins, enzymes, lipids, and other macromolecules. Lipofuscin is produced from secondary products of membrane lipids peroxidation reacting with amino groups of phospholipids, proteins, etc. Lipofuscin is considered a less specific marker of lipid peroxidation than malondialdehyde [65]. It is stored in lysosomes, decreasing their phagocytic activity and ability to degrade the nonefficient and damaged mitochondria, a phenomenon accompanying the aging process [66–68]. Lipofuscin is a fluorescent complex mixture composed of highly oxidized cross-linked macromolecules (proteins, lipids, and sugars) with multiple metabolic origins [69]. The nature and structure of LPS complexes vary among tissues and show temporal heterogeneity in oxidized proteins (30–70%), lipids (20–50%), metals cations (2%), and sugar residues content. Due to its polymeric and highly cross-linked nature, LPS cannot be degraded or cleared by exocytosis. It accumulates within the lysosomes and cell cytoplasm of long-lived postmitotic and senescent animal cells [69]. To the best of our knowledge, there are barely any studies linking lipofuscin concentration with osteoarthritis and joint replacement in humans. Moreover, this subject was never analyzed in the context of lipofuscin serum level and its changes after physical rehabilitation. This study documents a significant reduction in LPS levels in hemolysates of the patients after a 21-day systemic physical activity. In our previous study, serum lipofuscin concentration significantly decreased after the 21-day general rehabilitation program. We also report that lipofuscin in erythrocytes decreases after the 21-day general rehabilitation program. We assume that high lipofuscin concentration is associated with high oxidative stress resulting from precedent joint damage and recent surgery. The rehabilitation program positively affected, meaning reduced, the oxidative stress in the patients and contributed to the subsequent reduction of lipofuscin concentration, as expected from literature analysis [40, 70].

Oxidation changes membrane permeability resulting in hemolysis which may reflect endovascular destruction of the erythrocytes. The extravascular mechanisms may include cell deformation and changes in antigenicity [71]. Lipid peroxidation causes polymerization of membrane components and decreases cells' deformability [72]. Lipid oxidation products, such as oxidized cholesterol and oxidized unsaturated fatty acyl groups of phospholipids, may affect membrane bilayer structure and function. Phospholipids uptaken from plasma repair the oxidized lipids of the erythrocytes membrane since they cannot be synthesized *de novo*. Overall, lipid peroxidation decreases membrane fluidity [73]. The surface receptor molecules that allow cells to respond to hormones and cytokines and are involved in the maintenance of correct ion balance within the cells can be inactivated during lipid peroxidation [74]. Redox imbalance and oxidative stress-induced membrane shedding increase the cholesterol/phospholipids ratio in the erythrocyte membrane, decreasing membrane fluidity [75] and consequently elevating osmotic resistance [76–78]. Higher cholesterol level in the erythrocyte membrane affects its mechanical properties [79, 80]. During erythrocyte physiological aging, senescent erythrocytes show an increased cholesterol/phospholipids ratio and subsequent increased membrane osmotic resistance [77]. Increased cholesterol/phospholipids ratio is observed in various pathological conditions such as diabetes [75], chronic alcoholism [81], and multiple sclerosis [82]. The significant elevations in HDL-C and LDL-C serum levels were noted after a 21-day physical rehabilitation [40]. The observed changes may be related to modulation of the erythrocytes' membrane fluidity that enhances its functionality. The cholesterol content in the erythrocyte membrane reflects the blood cholesterol levels of high- and low-density lipoproteins [83]. The increase in the cholesterol content, until a critical level, stabilizes the erythrocyte membrane and keeps its fluidity critical for its functions. Beyond this critical level, the increased cholesterol content decreases the membrane's fluidity and impairs its functions [83]. HDL plays a major role throughout the body by removing excess cholesterol from membranes, so they gain greater stability, but altogether, it ensures the critical flow necessary to perform the membrane functions. In our previous paper, we observed the increased HDL serum levels. We concluded that it indicated that the mechanisms removing the excess of cholesterol are more efficient in the membranes of extrahepatic tissues and cells, and they ensure the membrane critical fluidity and promote the so-called reverse transport of cholesterol to the liver [40, 83]. Our study showed a reduction of cholesterol levels accompanied by reduced LPS levels. We consider that the changes result from the protective mechanism stimulated by controlled physical activity of the patients related to the 21-day general rehabilitation program.

The suppressor of tumorigenicity 2 (ST2) is expressed on many different cells, mostly of hematopoietic origin, where IL-33 induces the production of cytokines and chemokines, cell activation, or chemotaxis [84–88]. Like other members of the IL-1 receptor family, ST2 also exists in a soluble form (sST2), generated by alternative mRNA splicing. This soluble form acts as a decoy receptor and inhibits IL-33 signaling [89]. Elevated sST2 concentrations have been reported in the serum of patients suffering from various disorders, including systemic lupus erythematosus, atopic dermatitis, asthma, trauma, septic shock, and myocardial infarction. Several studies suggested that IL-33 and ST2 are involved in the inflammatory process that leads to arthritis. It is known that IL-33 and ST2 are expressed in the synovium of patients with rheumatoid arthritis [90–92]. To date, only a few studies analyzed the relationship between serum ST2 levels and osteoarthritis severity and development. Sacitharan et al. [93] studied tissues from OA patients and reported an upregulation of IL-33 in the synovial fluid and both IL-33 and its receptor ST2 in chondrocytes. The *in vitro* experiments on human chondrocytes cells showed that exogenous IL-33 stimulated human chondrocytes to produce cartilage-degrading proteases. Also, in the experimental mice model, exogenous IL-33 augmented the disease in animals with experimentally induced OA by destabilization of the medial meniscus (DMM) [93]. Interleukin- (IL-) 33 is a type of cytokine, which is a ligand for ST2, and belongs to the IL-1 receptor (IL-1R) family [94]. Li et al. [94] investigated the possible pathophysiological role of IL-33/sST2 in ankylosing spondylitis (AS). The authors reported that the serum levels of IL-33/sST2 were remarkably higher in the patients with AS than the healthy groups. Elevated levels of IL-33/sST2 were detected in the patients with peripheral arthritis, sST2 was higher in the patients with hip involvement, and IL-33/sST2 may regulate the immunological or inflammatory process of AS [94]. He et al. [95] showed that IL-33 and ST2 are elevated in human and murine OA. Moreover, neutralizing IL-33 and ST2 reduced cartilage degradation and pain *in vivo* and was associated with a marked decrease in the production of cartilage-degrading proteases alongside an increased expression of chondrogenic markers [95]. Pharmacological usage of monoclonal antibodies to block either IL-33 or ST2 helped diminish the pain and joint damage in mice with DMM-induced OA. It suggests that IL-33 can potentially be a future therapeutic target for OA [95]. In the presented study, we assessed the ST2 parameter in order to determine whether the tested biomarkers could help with identifying patients who benefited the most from the general 21-day rehabilitation process. So far, no scientific reports have studied ST2 in the context of physical rehabilitation of patients with OA. Our study shows a significant reduction in ST2 concentration in the serum of patients after knee or hip replacement in the course of osteoarthritis subjected to the 21-day general rehabilitation program. We may conclude that ST2 is one of the many inflammation-related parameters that are altered in patients with OA who underwent hip or knee replacement and the general rehabilitation program. The general rehabilitation program reduces the inflammatory processes measured by the ST2 parameter.

## 5. Conclusions

General rehabilitation is an effective, natural, and therapeutic procedure reducing the levels of oxidative stress markers such as LPS, as well as cholesterol and ST2 in patients, who underwent knee and hip replacement in the course of osteoarthritis. Individually designed, regular physical activity is an essential element of the postoperative protocol, which improves the redox balance and helps patients recover after the surgery effectively.

## Figures and Tables

**Table 1:** Relationship between gender, age, and type of joints affected by osteoarthritis in patients included in a 21-day general rehabilitation program after knee or hip surgical replacement. The results are presented as mean ± SD or median.

		Age (years)	*t*	*p*
Gender	Women	59.3 ± 5.4	0.179	0.859
	Men	58.9 ± 8.3		
Operated joint	Knee	59.0 ± 4.5	0.028	0.978
	Hip	59.1 ± 7.9		

**Table 2:** Change in the general health indicators in the blood of patients after knee or hip replacement subjected to the 21-day general rehabilitation program. The results are presented as a number (%) of cases.

Parameter	Amelioration (*N* (%))	No change (*N* (%))	Deterioration (*N* (%))
CRP (mg/L)	33 (80%)	2 (5%)	6 (15%)
ESR (mm/h)	27 (66%)	1 (2%)	13 (32%)
Glucose (mmol/L)	13 (32%)	26 (63%)	2 (5%)

Abbreviations: CRP: C-reactive protein, ESR: erythrocyte sedimentation rate.

**Table 3:** Oxidative stress markers levels in hemolysates and lipofuscin (LPS), cholesterol, and suppression of tumorigenicity 2 (ST2) levels in serum of patients (*n* = 36) after knee or hip replacement before and after a 21-day general rehabilitation program. The results are presented as mean ± SD or median and lower–upper quartile.

Oxidative stress markers	Before rehabilitation	After rehabilitation	Δ = after–before	*t*	*p*
CAT activity (IU/g Hb)	700.5 (649.2-748.4)	678.8 (650.0-750.8)	—	—	0.643
GR activity (IU/g Hb)	9.3 ± 1.7	9.2 ± 2.1	—	0.34	0.734
SOD activity (NU/mg Hb)	232.0 ± 19.2	295.5 ± 31.3	63.5	15.88	<0.001
GPx activity (IU/g Hb)	60.0 ± 9.0	71.2 ± 10.9	8.2	8.23	<0.001
GST activity (IU/g Hb)	0.41 ± 0.09	0.42 ± 0.11	—	0.95	0.349
LPS (RF)	314.9 ± 102.7	260.2 ± 48.9	-86.6	3.74	<0.001
Cholesterol (mg/dL)	234.0 ± 35.0	217.6 ± 31.5	-16.4	5.10	<0.001
ST2 (ng/mL)	0.69 ± 0.11	0.51 ± 0.13	-0.18	8.99	< 0.001

Abbreviations: CAT: catalase, GPx: glutathione peroxidase, GR: glutathione reductase, GST: glutathione S-transferase, Hb: hemoglobin, LPS: lipofuscin, RF: relative lipid extract fluorescence, SOD: total superoxide dismutase, ST2: suppression of tumorigenicity 2 marker.

## Data Availability

Data are available on request due to privacy/ethical restrictions.
